# Efficacy of iron fortification compared to iron supplementation among Vietnamese schoolchildren

**DOI:** 10.1186/1475-2891-5-32

**Published:** 2006-12-05

**Authors:** Huong Thi Le, Inge D Brouwer, Jan Burema, Khan Cong Nguyen, Frans J Kok

**Affiliations:** 1 Nutrition Department, Hanoi Medical University, 1 Ton That Tung St. Dong Da, Hanoi, Vietnam; 2 Division of Human Nutrition, Wageningen University, P.O.Box 8129, 6700 EV Wageningen, The Netherlands; 3 National Institute of Nutrition, Hanoi. 48B Tang Bat Ho St. Hanoi, Vietnam

## Abstract

The effect of iron fortification is generally assumed to be less than iron supplementation; however, the magnitude of difference in effects is not known. The present study aims to compare the efficacy of these two strategies on anaemia and iron status. After screening on low Hb, 425 anaemic children in six primary schools in Tam Nong district of Phu Tho province were included in a randomized, placebo-controlled trial comparing two groups receiving iron fortified instant noodles or iron supplementation for 6 months and a control group, with children in all groups having been dewormed. Blood samples were collected before and after intervention for haemoglobin, serum ferritin (SF), serum transferrin receptor (TfR), C-reactive protein (CRP), and haemoglobinopathies analysis. Regression analysis was used to assess the effect of iron fortification and iron supplementation on haemoglobin concentration, SF, TfR, body iron, and anaemic status as outcome variables. The improvement of haemoglobin, SF, and body iron level in the group receiving iron fortification was 42% (2.6 g/L versus 6.2 g/L), 20% (23.5 μg/L versus 117.3 μg/L), and 31.3% (1.4 mg/kg versus 4.4 mg/kg) of that in the iron supplementation group. The prevalence of anaemia dropped to 15.1% in the control group, with an additional reduction of anaemia of 8.5% in the iron supplementation group. The additional reduction due to iron fortification was 5.4%, which amounts to well over 50% of the impact of supplementation. In conclusion, the efficacy of iron fortification based on reduction of prevalence of anaemia, and on the change in haemoglobin level, is about half of the maximum impact of supplementation in case of optimal compliance. Thus, in a population of anaemic children with mild iron deficiency, iron fortification should be the preferred strategy to combat anaemia.

## Background

Anaemia is a significant public health problem in Vietnam. The 2000 National Nutrition Risk Factor Survey in Vietnam showed an anaemia prevalence of 34% in children under five and 25% in women [1]. No nationally representative data are available on the prevalence of anaemia among primary schoolchildren in Vietnam; however, a few local studies show an anaemia prevalence of approximately 30% [2,3]. Iron deficiency is considered as the major cause of anaemia, due to low intake and bioavailability of iron in the diet [4,5]. The National Nutrition Survey shows that the mean iron intake of Vietnamese people, which is mainly non-haeme iron, only reaches 72% of the RDA [6]. While iron supplementation in itself is highly effective in reducing iron deficiency anaemia, the implementation has been characterized by low coverage (15–20%) and non compliance [1].

Food-based strategies are recommended as long-term interventions to address the malnutrition problem in the country [7]. Although it is generally accepted that the increase of consumption of animal products would increase iron intake in the long term, the consumption of animal products in developing countries is sincerely hampered by low socio-economic status [8]. Food fortification is often suggested as one of the most effective and sustainable strategies for increasing iron intake in the general population [4].

Studies on the effect of iron supplements [9-13] or iron-fortified foods [14-18] on indicators of iron deficiency have been carried out, but few studies compare the effect of iron fortification with iron supplementation on the improvement of iron and anaemia status. It is generally known that fortification is less effective than supplementation due to differences in iron dose and the bioavailability of iron [19]. However, the extent of the differences in effect is unknown. In a previous study, Baltussen *et al*. suggested that fortification would be 50% less effective than supplementation, but this assumption was not based on an intervention study [19].

The aim of the present study is to compare the effect of iron fortification and iron supplementation on the changes in haemoglobin and iron status in order to assist public health nutritionists in making an informed choice for developing an appropriate strategy to combat iron deficiency and anaemia among schoolchildren in rural Vietnam.

## Subject and methods

### Study design and population

The study was implemented from November 2004 to May 2005 in six primary schools in Tam Nong district, Phu Tho province, situated 90 km from Hanoi. Selection was based on the high prevalence of anaemia and the absence of interventions to control iron deficiency anaemia in schoolchildren. Children recruited into the study were in grade one to grade three with haemoglobin concentrations < 110 g/L but not <70 g/L in an initial haemoglobin-screening study. We excluded children with Hb level less than 70 g/L because these children were considered as severely anaemic and received treatment immediately.

**Figure 1:**
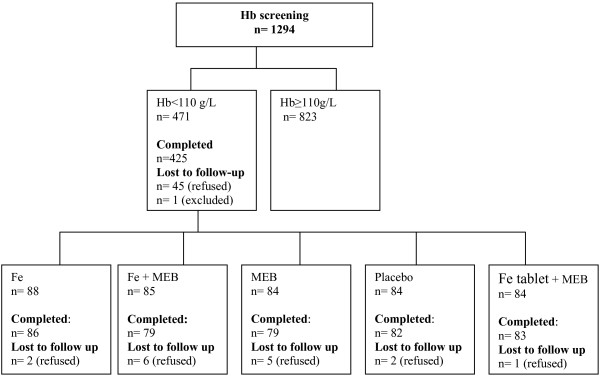
Study profile: initial screening to enroll anaemic children in the study, followed by a 6 month intervention.

The study concerns a randomized, placebo-controlled double blind parallel trial with a 2 × 2 factorial design plus standard treatment (iron supplementation and de-worming) and an intervention period of six months. A total of 425 eligible children were randomly assigned to one of five groups (85 per group) receiving: I) iron-fortified noodles and mebendazole (Fe+MEB); II) noodles without iron fortificant and mebendazole (MEB); III) iron-fortified noodles and placebo (Fe); IV) noodles without iron fortificant and placebo (placebo); and V) iron supplementation and mebendazole (Fe tablet+MEB) (Figure 1). Randomization was carried out by a researcher from the Division of Human Nutrition, Wageningen University, The Netherlands, who did not know the children and could not introduce bias in the randomization. Stratified randomization was applied based on the Hb levels and age of the children to ensure equal distribution of Hb and age across groups. Sample size was assessed to achieve a statistical power of 95% at an alpha level of 0.05, based on the expectation that the treatment groups would have a haemoglobin concentration at the end of the study of 5 g/L higher than the control group, assuming a standard deviation of 11 g/L [2]. Furthermore, it was anticipated that some children would drop out during the intervention; therefore, the sample size was increased by 10% at the beginning of the study.

In this article, we only concentrate on the effect of the iron fortified noodles (Fe+MEB) compared to that of the standard treatment (Fe tablet+MEB). For this reason, three groups were included in this analysis: (Fe+MEB), (Fe tablet+MEB), and (MEB) as the control group. The effect of iron-fortification and de-worming on iron and anaemia status of schoolchildren is discussed in another paper [20]. Children were invited to participate in the study, and their parents signed written consent forms. The study was approved by the Scientific Committee of the National Institute of Nutrition and the Ethics Committee of Hanoi Medical University – Ministry of Health.

### Products and field procedures

Fortified instant noodles were produced by the Hanoi Food Company. Noodles were fortified with a water soluble, highly bioavailable iron compound (NaFeEDTA: Ferrazone^®^, Akzo Nobel Chemicals Pte Ltd Arnhem) to a fortified level of 10.7 mg iron per 52 gram of noodles calculated based on the JECFA 1974 recommendation of an acceptable daily intake of 2.5 mg EDTA/kg body weight and an average body weight of 29 kg [21]. Before the intervention, retention of iron after production and preparation of fortified instant noodles was checked in laboratories at the National Institute of Nutrition in Hanoi, Wageningen University, and Akzo Nobel Chemicals Pte Ltd in Arnhem. Capillary zone electrophoresis analysis [22] showed that 70% of the NaFeEDTA dissolves within 5 minutes into the soup independent of extraction time. No degradation products of NaFeEDTA were found.

Noodles were prepared in schools by caretakers trained by the field staff and given to children at break time (9:00 am) five days per week during six months under the supervision of teachers and field staff. Children were encouraged to consume all of the noodles and liquid soup. Mebendazole 500 mg was given to children at the beginning of the intervention as well as three months following the intervention. Children, caretakers, teachers, and researchers were not aware of the type of treatment which was given. Iron supplementation in the form of ferrous fumarate 200 mg (equal to 65 mg elemental iron) was taken with a glass of water at break time every school day (five days a week). Ingestion of the supplements was supervised by a teacher and field staff and then recorded in a monitoring book.

### Data collection

Capillary blood samples were taken from the children's fingers during screening for haemoglobin measurement by cyanmethaemoglobin method. Venous blood (5 ml) was collected in the morning at baseline (T0) and after intervention (T6); 20 μl whole blood was pipetted immediately before coagulating into a tube containing 5.0 ml of Drabkin's reagent with a Sali pipette for haemoglobin measurement. An aliquot of whole blood was taken for analyzing haemoglobinopathies. The remaining blood was allowed to clot for 30 minutes at room temperature, centrifuged at 3000 × g for 15 minutes, and transferred to five plastic labeled vials (Eppendorf tubes 0.5 ml). The vials were stored in a box protected from sunlight, put into an icebox for transfer to the laboratories, and kept at -80°C for serum ferritin (SF), serum transferrin receptor (TfR), and C-reactive protein (CRP) analysis at the end of the intervention.

For assessment of intestinal parasite infection before and after the intervention, containers for collection of stools were distributed to each class, and children were asked to collect and deliver a sample of their faeces to school the next day. In case some children were unable to return a sample, one of the field workers returned the next day to collect the rest of the samples.

### Laboratory analysis

Haemoglobin concentration was measured in whole blood within 12 hours of sampling by cyanmethaemoglobin method using Sigma KIT in the Tam Nong District Health Centre. The CV of intra-assays and inter-assays was 4.0 ± 1.2% and 5.0 ± 2.0 % respectively. SF, TfR, and CRP analyses were carried out at the same time for both samples of baseline and after intervention at the National Institute of Nutrition and the laboratory of Hanoi Medical University in May and June 2005. Concentrations of SF and TfR were analyzed by an Enzyme-Linked Immuno Sorbent Assay (ELISA) method (Ramco Laboratories, Inc, Houston, TE, Catalogue numbers S-22 and TF-94), with inter-assay variability of 4–7% and 4–8%, respectively. Serum CRP was measured by nephelometry using Epress plus, with an inter-assay variability of 4–8 %. A 10% subsample was re-examined for quality control. Haemoglobinopathies analysis was performed using the Variant Beta-Thalassemia Short program (Bio-Rad laboratories Inc, Hercules, CA) within 24 hours of sampling in the Children's Hospital, Hanoi, Vietnam. Stools samples were examined before and after intervention using the Kato-Katz Technique – a cellophane faecal thick smear method [23]. Hookworm, *Trichuris*, and *Ascaris *eggs were counted. A 10% subsample of smears was re-examined for quality control.

### Data analysis

Anaemia was defined as haemoglobin concentrations <115 g/L [24]. Iron deficiency was defined as SF concentrations <12 μg/L [24], and tissue iron deficiency was defined as TfR concentration >8.5 mg/L [25]. Body iron content was calculated using the following formula: body iron (mg/kg) = -(log(TfR/SF ratio) – 2.8229)/0.1207 [26]. CRP concentration was considered to be elevated when ≥ 8 mg/L [27]. Haemoglobin type was determined in each subject on the basis of haematological indexes: HbAA (normal haemoglobin type), HbF, HbA2 (Beta thalassemia), Hb AE (trait for haemoglobin E disease) or HbEE (haemoglobin E disease). The severity of intestinal worm infections was expressed by the number of eggs/g faeces using the WHO classification system [28]. We excluded all children with thalassemia and haemoglobin E (HbF, HbA2, HbAE) (n = 15) and elevated CRP (n = 5) from the analysis to prevent confounding.

Data was entered into the computer, cleaned and managed using Epi Info version 6, [29] and analyzed using SPSS 11.0 for windows (SPSS Inc., Chicago IL, USA)[30]. Data was checked for normality by visual observation. One-way ANOVA was used to determine differences in haemoglobin concentration and other biochemical indicators between groups. Paired *t*-tests were used to assess the difference in haemoglobin and other biomarkers within the group before and after intervention. Chi-square tests and Wilcoxon tests were used to assess the differences between and within groups according to the proportion of anemia and other indicators.

To assess the differential effect of iron fortification as compared to iron supplementation on indicators of iron status, we compared children with iron fortification only, children with iron supplementation only, and a control group without fortification or supplementation with respect to their change in haemoglobin concentration, SF, TfR, and body iron, respectively. This was done by using multiple linear regression analysis, including two dummy variables for the intervention groups, and accounting for differences in baseline value, sex and age distribution.

## Results

At the baseline, the mean age of children was 87.3 ± 10.3 months. The three groups did not significantly differ in age, haemoglobin concentration, iron status (SF, TfR, and body iron) (Table 1) or parasite infection (Table 2). The prevalence of iron deficiency was very low as 0.9% of children showed SF concentration below 12 μg/L, and 3.2% of children showed TfR above 8.5 mg/L. Mean body iron was around 6.3 mg/kg body weight (Table 1). As much as 66%, 71% and 9% of children were infected with *Ascaris, Trichuris*, and hookworm, respectively (Table 2).

**Table 1:** Baseline characteristic of Vietnamese schoolchildren by group after random assignment (n = 221)

	Fe + MEB (n = 72)	Fe tablet + MEB (n = 76)	MEB (Control) (n = 73)
Male sex (%)	48.6	51.3	46.6
Age (month)^1^	87.3 ± 11.6	86.4 ± 9.8	87.9 ± 10.2
Haemoglobin (g/L)^1^	107.6 ± 6.9	108.4 ± 6.7	108.9 ± 6.5
SF (μg/L)^2^	46.8 (33.3 – 66.4)	54.2 (39.7 – 72.4)	54.5 (37.8 – 79.7)
TfR (mg/L)^1^	6.0 ± 1.3	5.9 ± 1.1	6.2 ± 1.4
Body iron (mg/kg)^1^	6.0 ± 2.3	6.6 ± 1.9	6.3 ± 2.7
SF < 12 μg/L (%)	1.4	0	1.4
TfR > 8.5 mg/L (%)	2.8	1.3	5.5

^1 ^Mean ± SD

^2 ^Geometric mean (25 and 75 percentile)

Fe + MEB: Iron-fortified noodles and mebendazole

Fe tablet + MEB: Iron supplementation and mebendazole

MEB: Mebendazole

**Table 2:** Change in haemoglobin, iron status indicators, and worm infection after 6 months of intervention among Vietnamese schoolchildren

	Fe + MEB (n = 72)	Fe tablet + MEB (n = 76)	MEB (Control) (n = 73)
Change in Haemoglobin (g/L)^1^	17.8 ± 7.6^2^	21.2 ± 10.7^2^	14.5 ± 8.5^2^
Change in SF (μg/L)^1^	18.5 ± 30.9^2^	111.7 ± 76.5^2^	-6.5 ± 27.1
Change in TfR (mg/L)	-0.4 ± 0.9^2^	-0.8 ± 0.9^2^	-0.4 ± 0.9^2^
Change in Body iron (mg/kg)^1^	1.5 ± 1.9^2^	4.2 ± 1.9^2^	-0.1 ± 1.6
Anaemia (%)			
T0	83.3^3^	84.2^3^	83.6^3^
T6	9.7	6.6	15.1
SF <12 μg/L (%)			
T0	1.4	0	1.4
T6	0	0	0
TfR >8.5 mg/L (%)			
T0	2.8	1.3	5.5
T6	2.8	0	0
*Ascaris *(%)			
T0	62.5	67.1	68.5
T6	41.7^5^	47.4^4^	41.1^4^
*Trichuris *(%)			
T0^1^	77.8	72.4	63.0
T6^5^	15.2^3^	47.4^3^	47.9^5^
Hookworm (%)			
T0	8.3	10.5	8.2
T6	0^5^	1.3^5^	1.4^5^

^1 ^Significant difference between group (one-way ANOVA): ^1^p < 0.001;

^2 ^Significant difference within group before and after intervention (t test); ^2^p < 0.001;

^3 ^Significant difference within group before and after intervention (McNemar); ^3^p < 0.001; ^4^p < 0.01;^5^p < 0.05;

Fe + MEB: Iron-fortified noodles and mebendazole

Fe tablet + MEB: Iron supplementation and mebendazole

MEB: Mebendazole

Haemoglobin concentration increased in all three groups; however, a larger significant increase was seen in the group receiving iron supplementation: 21.2 ± 10.7 g/L compared to 17.8 ± 7.6 g/L and 14.5 ± 8.5 g/L in iron fortified and control groups (Table 2). Prevalence of anaemia significantly decreased in all three groups but to a larger extent in the two groups receiving iron fortified noodles and iron supplementation (after six months of intervention prevalence was only 9.7% and 6.6%, respectively) than in the control group where the prevalence dropped down to 15.1% (Table 2).

SF concentration increased significantly in the two groups receiving iron fortification and iron supplementation (18.5 ± 30.9 μg/L and 111.7 ± 76.5 μg/L respectively) compared to the control group where SF concentration even decreased (-6.5 ± 27.1 μg/L) (Table 2). TfR concentration was very limited improved after six months of intervention in all three groups; however, the group receiving iron supplementation showed largest improvement (-0.8 ± 0.9 mg/L) compared to iron fortification and control groups (-0.4 ± 0.9 mg/L and -0.4 ± 0.9 mg/L). There were no significant differences between groups (Table 2). Body iron significantly increased in the two groups receiving iron fortification and iron supplementation (1.5 ± 1.9 mg/kg and 4.2 ± 1.9 mg/kg respectively) compared to the control group (-0.1 ± 1.6 mg/kg). Prevalence of *Ascaris, Trichuris *and hookworm infection fell significantly in all three groups (Table 2).

The additional change in haemoglobin in the intervention groups as compared to the control group was estimated by taking into account the baseline Hb value in the regression model, in addition to accounting for age and sex (Table 3). Similar differential changes were calculated for SF, TfR, and body iron. The additional improvement of haemoglobin, SF, and body iron level in the group receiving iron fortification was 42% (2.6 g/L compared to 6.2 g/L); 20% (23.5 μg/L compared to 117.3 μg/L) and 31% (1.4 mg/kg compared to 4.4 mg/kg) of that in the iron supplementation group (Table 3). In the control group, a reduction of anaemia to 15.1% was observed after 6 months of intervention. In the iron supplementation group anaemia dropped down to 6.6%, which was an additional reduction of 8.5%. In the group treated with iron fortification a smaller additional reduction of 5.4% (down to 9.7%) could be achieved, which nevertheless amounted to more than 50% of the impact of supplementation. (Table 2).

**Table 3:** Differential change in haemoglobin, SF, TfR, and body iron during intervention in two intervention groups compared to the control group, from 4 multiple linear regression models

	Intervention group			
	
Outcome variables	Iron fortification*	*p*	Iron supplementation*	*p*
Haemoglobin (g/L)^1^	2.59 (-0.22 – 5.40)	0.07	6.19 (3.42 – 8.96)	0.001
SF (μg/L)^2^	23.5 (6.82 – 40.25)	0.006	117.3(100.86 – 133.64)	0.001
TfR (mg/L)^3^	-0.04 (-0.32 – 0.23)	0.76	-0.51 (-0.78 – -0.24)	0.001
Body iron (mg/kg)^4^	1.37(0.85 – 1.89)	0.001	4.37 (3.86 – 4.88)	0.001

^1 ^Adjusted for Hb baseline, sex and age

^2 ^Adjusted for SF baseline, sex and age

^3 ^Adjusted for TfR baseline, sex and age

^4 ^Adjusted for body iron baseline, sex and age

* Regression coefficients (95% CI)

## Discussion

Results from the present study show that in anaemic schoolchildren, iron fortification was 58% (based on change in haemoglobin level), 80% (based on SF level), and 69% (based on body iron) less effective than iron supplementation. However, the risk of remaining anaemic was reduced considerably in all study groups, and fortification appeared to have a beneficial effect on the additional reduction due to treatment of about half as much as that of supplementation.

Data collection in our study was carried out carefully. Blood samples were collected, transported, and stored under standard conditions. Serum samples before and after intervention were analyzed at the same time after intervention to avoid variation between different measurements. In-house quality control was carried out regularly during serum analysis at the laboratories. Randomization was successful, as the groups were comparable in the key indicators at baseline. De-worming was effective as shown by a significant reduction of intestinal parasite infection in all three groups.

In the present study, the control group also improved haemoglobin and anaemia status after 6 months of the intervention which might be explained by the effect of de-worming. However, although de-worming reduced worm infection prevalence, no effect of de-worming on the anaemia and iron status could be established. The explanation for that was that although two thirds of the children were infected with *Ascaris *and/or *Trichuris*, and about 8% infected with hookworm, it turned out that only 27% and 2% among infected children showed severe infection with *Ascaris *or *Trichuris*, respectively. The hookworm infection was 'light' for most of the cases [20]. A previous analysis of all five study groups suggested that in the absence of other major causes of anaemia (such as vitamin A deficiency, malaria, worm infection and haemoglobinopathies), probably chronic inflammation could have played a role, but this needs to be further addressed [20].

A large part of the selected school children was anaemic at baseline (84%) but showed surprisingly few iron deficiency as indicated by the low prevalence of elevated SF and TfR indicators (0.9% and 3.2%). However, an improvement of haemoglobin levels in an anaemic population through iron supplementation is commonly seen as an indicator of the presence of iron deficiency [31], and the improvement of haemoglobin levels in our anaemic population still indicates possibly mild iron deficiency although not confirmed by the SF and TfR levels.

After adjustment for differences in baseline values of indicators of iron deficiency, the improvement of haemoglobin concentration was 2.6 g/L and 6.2 g/L in excess of the increase in the control group. The accompanying additional reduction of anaemia was 5% to 9% in the treated groups. The estimated additional improvement of haemoglobin and reduction of anaemia in our study sample is slightly smaller than found in other studies. In a study among anaemic Vietnamese women consuming daily 10 ml fish sauce containing 10 mg elemental iron from NaFeEDTA during 6 months, haemoglobin changed with 5.7 ± 10.3 g/L and -2.8 ± 8.7 g/L in the intervention and control group respectively [17]. A study in children 12–17 years with mild or moderate anaemia in Malaysia reports that after 22 weeks receiving weekly iron supplementation of 60 mg elemental iron (as ferrous sulfate) and 3.5 mg folate, there was an improvement of haemoglobin concentration of 21.4 g/L compared to 9.3 g/L in the control group receiving only 3.5 mg folate [32]. A review of fortification and supplementation studies in Indonesia demonstrates that iron supplementation can reduce anaemia prevalence in pregnant women by 20 to 25 % and iron fortification (adding 10 mg of elemental iron) can reduce anaemia by 20% among those consuming the fortified foods [33]. As the amount of iron absorbed, and hence the magnitude of improvement of haemoglobin concentration and reduction of anaemia, depends on the iron and anaemia status of the individual [32], the lower improvement in our study may indicate a mild iron deficiency compared to the study in Vietnamese women (69.9% with iron deficiency anaemia) [17].

On the basis of the change in haemoglobin in this population with anaemia but mild iron deficiency, iron fortification is 58% less effective than iron supplementation. This reduced efficacy can be explained by the difference in the given amount of iron being lower in the fortification than in the supplementation group. Most of the supplementation programs for women, school-age children or adolescents usually use 60 mg iron/day [34]. However, although in our study the daily amount of iron received from iron-fortified noodles (10.7 mg/day) is 6 times less than from iron supplementation (65 mg/day), the improvement of haemoglobin level in the group receiving iron fortification reaches almost half of the improvement seen in the iron supplementation group (2.6 g/L compared to 6.2 g/L) (Table 3). Also, the additional reduction in risk of being anaemic by iron fortification was about half as much as it was by supplementation. Iron stored, as indicated by SF, was 5 times higher in the group receiving supplementation than in the group receiving iron fortification (117.3 μg/L and 23.5 μg/L respectively). However, our study population were anaemic children with low prevalence of iron deficiency; therefore, the effect of iron fortification relative to iron supplementation may differ from a population with a high prevalence of iron deficiency.

Food fortification is often suggested as one of the most cost-effective and sustainable strategies for increasing iron intake in the general population [4,35]. We used NaFeEDTA as iron fortificant in our study. Besides the advantages of NaFeEDTA with regard to iron absorption and stability, the main disadavantage is its relatively high price compared with other fortificants like ferrous sulphate. The price of imported NaFeEDTA was $6/kg. In our study, the additional cost has been estimated to be $ 0.01/kg of instant noodles. This is affordable for people in the rural areas and is comparable to the fishsauce fortification program in Vietnam in which the additional cost of NaFeEDTA fortified fishsauces was $0.02/L [17,18].

In conclusion, the efficacy of iron fortification based on reduction of prevalence of anaemia, and on the change in haemoglobin level, is about half of the maximum impact of supplementation in case of optimal compliance. Thus, in a population of anaemic children with mild iron deficiency, iron fortification should be the preferred strategy to combat anaemia.

## Abbreviations used

SF, serum ferritin; TfR, serum transferrin receptor; CRP, C-reactive protein; (ELISA), Enzyme-Linked Immuno Sorbent Assay; (Fe+MEB), iron-fortified noodles and mebendazole; (MEB), noodles without iron fortificant, and mebendazole; (Fe), iron-fortified noodles and placebo; Placebo, noodles without iron fortificant and placebo; (Fe tablet+MEB), iron supplementation and mebendazole.

## Authors' contributions

HTL was responsible for all aspects of protocol development, study coordination, data collection, data analysis, and report writing. IDB was involved in protocol development, data analysis, report writing, and obtaining funds for the study. JB supported in statistical analysis. KCN was involved in the supervision of data collection. FJK was involved in protocol development, data analysis, reporting, and obtaining funds for the study.

## References

[B1] Ninh NX, Khan NC, Khoi HH (2001). Micronutrient deficiencies and strategies for their control in Vietnam. ln:20 years of prevention and control of Micronutrient Deficiency in Vietnam. Medical Publishing House, Hanoi, Vietnam.

[B2] Hoa DT (2002). Effect of fortified biscuit with vitamin A and iron on vitamin A status and anemia in primary school children in  sub- urban Vietnam.

[B3] Huong LT (1999). Nutritional status and related factors of primary school children in Hanoi and sub urban Hanoi.

[B4] Hurrell RF (1997). Preventing iron deficiency through food fortification. Nutr Rev.

[B5] Committee. INACGS, INACG (2002). Anemia, iron deficiency and iron deficiency anemia.

[B6] NIN/MOH (2000). Nutritive composition table of Vietnamese foods.. Medical Publishing House, Hanoi, Vietnam.

[B7] Vietnam MOH, MOH (2001). National Nutrition Strategy, period 2001-2010.

[B8] Hurrell RF, Lynch S, Bothwell T, Cori H, Glahn R, Hertrampf E, Kratky Z, Miller D, Rodenstein M, Streekstra H, Teucher B, Turner E, Yeung CK, Zimmermann MB (2004). Enhancing the Absorption of
Fortification Iron. Int J Vitam Nutr Res.

[B9] Thu BD, Schultink W, Dillon D, Gross R, Leswara ND, Khoi HH (1999). Effect of daily and
weekly micronutrient supplementation on
micronutrient deficiencies and growth in young
Vietnamese children.. Am J Clin Nutr.

[B10] Ekstrom EC, Kavishe FP, Habicht JP, Frongillo EA, Rasmussen K, Hemed L (1996). Adherence
to iron supplementation during pregnancy in
Tanzania: Determinants and haematological
consequences.. Am J Clin Nutr.

[B11] Suharno D, West CE, Karyadi D, Hautvast JG, Muhilal (1993). Supplementation with
vitamin A and iron for nutritional anaemia in
pregnant women in West Java, Indonesia.. The Lancet.

[B12] Allen L, Rosado J, Casterline J, Lopez P, Munoz E, Garcia O, Martinez H (2000). Lack of
haemoglobin response to iron supplementation in anaemic Mexican preschoolers with multiple
micronutrient deficiencies. Am J Clin Nutr.

[B13] Paracha PI, Khan SM, Ahmad I, Nawab G (1993). Effect of iron supplementation on biochemical indices of iron status in selected pre-adolescent schoolgirls in North West Frontier Province, Pakistan. Asia Pacific J Clin Nutr.

[B14] Layrisse M, Chaves JF, Mendez-Castellano H, Bosch V, Tropper E, Bastardo B, Gonzalez E (1996). Early response to the effect of iron
fortification in a Venezuelan population.. Am J Clin Nutr.

[B15] Viteri FE, Alvarez E, Batres R, Torún B, Pineda O, LA LAM, Sylvi J (1995). Fortification of sugar
with iron sodium ethylenediaminotetraacetate
(FeNaEDTA) improves iron status in semirural
Guatemalan populations.. Am J Clin Nutr.

[B16] Hurrell RF, Furniss DE, Burri J, Whittaker P, Lynch SR, Cook JD (1989). Iron fortification of
infant cereals: a proposal for the use of ferrous
fumarate or ferrous succinate.. Am J Clin Nutr.

[B17] Thuy PV, Berger J, Cavidsson L, Khan NC, Lam NT, Cook JD, Hurell RF, Khoi HH (2003). Regular consumption of NaFeEDTA-fortified fish sauce improves iron status and reduces the prevalence of anemia in anemic Vietnamese women.. Am J Clin Nutr.

[B18] Thuy PV, Berger J, Nakanishi Y, Khan NC, Lynch S, Dixon P (2005). The use of NaFeEDTA - Fortified fish sauce is an effective tool for controlling iron deficiency in women of childbearing age in rural Vietnam. J Nutr.

[B19] Baltussen R, Knai C, Sharan M (2004). Iron fortification and supplementation are cost-effective interventions to reduce iron deficiency in four subregions of the world.. J Nutr.

[B20] Huong LT, Brouwer ID, Khan NC, Jan B, Kok FJ (2005). Iron fortification, not de-worming improves anaemia  of  schoolchildren in rural Vietnam. not public yet.

[B21] FAO, WHO (1998). Requirement of Vitamin A, iron, folate and vitamin B12.. FAO Food and nutrition series No32.

[B22] Sheppard L, Henion J (1997). Quantitative capillary electrophoresis/Ion spray tandem mass spectrometry determination  of EDTA in human plasma and urine.. Analytical Chemistry.

[B23] WHO (1991). Basic laboratory methods in Medical Parasitology.. Geneva, Swetzeland: WHO,.

[B24] WHO (2000). Iron deficiency anaemia: Assessment, prevention and control..

[B25] Skikne BS, Flowers C, Cook JD (1990). Serum transferrin receptor: a quantitative measure of tissue iron deficiency. Blood.

[B26] Flowers CH, Skikne BS, James D. Cook (2003). The quantitative assessment of body iron. Blood.

[B27] Hoffbrand AV, Pettit JE, ed  (1993). Essential haematology.

[B28] series WHO, WHO (1987). Prevention and control intestinal parasitic infections. Report of a WHO Expert Committee.. Technical Report Series, No 749.

[B29] Dean AG, Dean JA, Coulombier D, Brendel KA, Smith DC, Burton AH, Dicker RC, Sullivan K, Fagan RF, Arner G (1995). Epi info version 6: a World - processing, database and statistics program for public health on IBM-Compatible Microcomputer.. Atlanta: Center for Disease Control and Prevention.

[B30] Field A, B.Wright D (2000). Discovering Statistics using SPSS for Windows.

[B31] Malope BI, MacPhail AP, Alberts M, Hiss DC (2001). The ratio of serum transferrin receptor and serum ferritin in the diagnosis of iron status. Br J Haematol.

[B32] Tee ES, Kandiah M, Awin N, Chong SM, Satgunasingam N, Kamarudin L, Dugdale AE, Viteri FE, Silvano Milani (1999). School-administered weekly iron-folate supplements improve
hemoglobin and ferritin concentrations in Malaysian adolescent girls. Am J Clin Nutr.

[B33] Sumarno I, Muhilal, Komari Review of surveys and supplementation studies of anaemia in Indonesia. http://www.unu.edu/unupress/food/8F171e/8F171E02.htm.

[B34] Stoltzfus RJ, Dreyfuss ML, Group(INACG) INAC, Washington DC Guidelines for the Use of Iron
Supplements to Prevent and Treat
Iron Deficiency Anemia.

[B35] Lotfi M, Venkatesh Mannar MG, Merx JHMR, van denHeuvel PN (1996). Micronutrient fortification of foods: current practices, research, and opportunities.

